# Ultrasound-Guided Analgesia in Cardiac and Breast Surgeries: A Cadaveric Comparison of SPIP Block with Single and Double Injections vs. DPIP Block

**DOI:** 10.3390/life15010042

**Published:** 2024-12-31

**Authors:** Carmelo Pirri, Debora Emanuela Torre, Astrid Ursula Behr, Veronica Macchi, Andrea Porzionato, Raffaele De Caro, Carla Stecco

**Affiliations:** 1 Department of Neurosciences, Institute of Human Anatomy, University of Padova, 35121 Padova, Italy; veronica.macchi@unipd.it (V.M.); andrea.porzionato@unipd.it (A.P.); rdecaro@unipd.it (R.D.C.); carla.stecco@unipd.it (C.S.); 2 Department of Cardiac Anesthesia and Intensive Care Unit, Cardiac Surgery, Ospedale dell’Angelo, 30174 Venice Mestre, Italy; deboraemanuela.torre@aulss3.veneto.it; 3 Department of Anesthesia and Intensive Care, ULSS 6 Euganea Padova, Camposampiero Hospital, 35012 Camposampiero, Italy; astrid.behr@aulss6.veneto.it

**Keywords:** analgesia, cardiac surgery, fascia, SPIP block, DPIP block, US-guided techniques

## Abstract

The evolution of regional anesthesia techniques has markedly influenced the management of postoperative pain, particularly in thoracic surgery. As part of a multimodal analgesic approach, fascial plane blocks have gained prominence due to their efficacy in providing targeted analgesia with minimal systemic side effects. Among these, the superficial intercostal plane (SPIP) block and deep parasternal intercostal plane (DPIP) block are of notable interest. The aim of this study was to investigate the dye spread to the anterior chest wall space and its spread pathway through anatomical morphometric analyses on cadavers for single-injection and double-injection SPIP blocks versus DPIP blocks. In both qualitative and quantitative evaluations, the single-injection SPIP block with 10 mL of dye demonstrated a broader and more extensive spread compared to the double-injection SPIP block, which used 5 mL of dye per injection site (*p* < 0.05), and the DPIP block with 10 mL of dye (*p* < 0.05). All the blocks had a positive correlation between the distances from the sternum border and the area of dye spread, suggesting that the crucial role of volume in fascial blocks is that it significantly affects the opening of the fascial compartment, enabling optimal spread of the anesthetic. Adequate volume facilitates proper spread and diffusion across the fascial plane, ensuring more comprehensive fascia coverage and thus enhancing the block’s effectiveness. Finally, precise volume management is key to maximizing both efficacy and safety.

## 1. Introduction

Postoperative pain management is a significant concern in both cardiac and breast surgeries, with effective analgesia critical for patient recovery and outcomes. Traditional systemic analgesics often lead to undesirable side effects. As a result, regional anesthesia techniques have gained prominence. Indeed, breast and cardiothoracic surgeries [1,2,3], as well as thoracic trauma [4], necessitate advanced techniques to achieve optimal postoperative analgesia, thereby promoting quicker recovery and facilitating early discharge. Clinical evidence suggests that regional anesthesia blocks present a more favorable side effect profile compared to traditional neuraxial techniques, providing advantages such as reduced opioid consumption, improved pain management, and diminished opioid-related side effects [5,6].

Chest wall blocks are among the most frequently utilized locoregional techniques in cardiothoracic surgery, breast surgery, and thoracic trauma analgesia, particularly for managing pain associated with rib fractures. There are several types of chest wall blocks, and their use is endorsed by Enhanced Recovery After Surgery (ERAS) protocols [7,8,9,10].

The terminology related to chest wall, abdominal wall, and paraspinal blocks has long been a source of confusion. In 2021, the ASRA-ESRA Delphi consensus significantly contributed to the standardization of this nomenclature [11]. The recent nomenclature, as established by the ASRA-ESRA Delphi consensus, has streamlined chest wall blocks into four main categories: deep serratus anterior plane block, superficial serratus anterior plane block, superficial parasternal intercostal plane block, and deep parasternal intercostal plane block. This standardization simplifies the classification and enhances the clarity of communication among practitioners. The previous nomenclature for parasternal blocks encompassed the pectointercostal fascial block and transversus thoracic muscle plane block. In the updated nomenclature, these are now referred to as the superficial parasternal intercostal plane (SPIP) block and the deep parasternal intercostal plane (DPIP) block, respectively.

The pectointercostal fascial block (PIFB), now known as the superficial parasternal intercostal plane (SPIP) block, was described by de la Torre et al. [12] in patients undergoing breast surgery. This technique targets the anterior cutaneous nerve, a branch of the intercostal nerve that provides sensory supply to the skin, soft tissue, and sternum. In the SPIP block, the injection is administered 2 cm lateral to the sternum, between the pectoralis major and the external intercostal muscles [12]. The transversus thoracic muscle plane block (TTMPB), now referred to as the deep parasternal intercostal plane (DPIP) block, is performed at the T3–T4 level, 1 cm lateral to the sternal border. The needle is inserted into the transversus thoracis muscle plane between this muscle and the internal intercostal muscle, with the local anesthetic deposited to avoid intravascular and intrapleural administration [13]. Both these blocks provide anterior chest wall analgesia by blocking the intercostal nerves. However, in the DPIP block, the injection of local anesthetic is closer to the anterior branches of the intercostal nerves [14,15]. The DPIP block, while effective, carries significant risks due to its proximity to the internal mammary artery (ITA), which can lead to serious complications like arterial injury and severe hemorrhage. Its effectiveness may also be compromised in procedures like coronary artery bypass grafting (CABG), where the internal thoracic artery is involved [16]. In contrast, the SPIP block, which is performed in a more superficial plane, poses a lower risk of vascular injury and does not interfere with ITA, making it potentially safer for CABG. However, these blocks have shown promise in clinical settings; their effectiveness can vary, and there are still several unresolved questions. The ongoing debate primarily centers around the actual spread of anesthetics determining a growing need to better understand their respective advantages, limitations, and clinical applications. Various studies have provided conflicting evidence, leading to differing opinions among researchers on this matter. To date, only two published cadaveric studies exist evaluating the SPIP block alone, one the SPIP block with a single and double injections and one SPIP block versus DPIP block [17,18]. Based on these considerations, the present study aimed to investigate the dye spread to the anterior chest wall space and its spread pathway through anatomical morphometric analyses on cadavers for single-injection and double-injection SPIP blocks versus DPIP blocks.

## 2. Materials and Methods

Two fresh cadavers (1 male/1 female) with a mean age of 72.3 ± 10 years and a BMI of 27.04 Kg/m^2^ were studied bilaterally (n = 12). Cadavers had no pathology, previous surgery, or trauma. Appropriate consent was obtained for cadavers donated to the Institute of Human Anatomy of the University of Padova according to the ‘Body Donation Programme’ of the Institute of Anatomy of the University of Padova [19]. This study was conducted in accordance with the principles of the Declaration of Helsinki. The authors state that every effort was made to follow all local and international ethical guidelines and laws that pertain to the use of human cadaveric donors in anatomical research [20,21]. The donors were specifically selected because they had no significant medical conditions that would affect the study’s results. None of the cadavers had a history of surgery involving the thorax based on medical record review and visual inspection of the specimens. Importantly, neither donor had been bedridden for an extended period, which could have led to changes in the trophism and integrity of the fascial planes due to prolonged supine or prone positioning.

### 2.1. Ultrasound-Guided Block Techniques

A single cardio-anesthesiologist expert in performing regional anesthesia (DET) conducted all blocks under direct ultrasound guidance to ensure precision and consistency in technique across all procedures. The procedures were performed on both sides of the chest. Each cadaver was positioned supine, and a preliminary ultrasound examination was conducted to ensure that there were no deviations in chest structures from normal human anatomy. This examination also confirmed that the relevant sono-anatomy was consistent with clinical observations in living individuals. For all the procedures, a high-resolution device (Edge II, Sonosite, FUJIFILM, Inc., Bothell, WA 21919, USA) with a frequency range of 6–15 MHz and a screen resolution of 1680 × 1050 pixels, with a linear transducer, was used. The US system used sound speed (c) of 1540 m/s. All procedures were carried out using a transducer cover sheet with standard colorless US gel inside. To ensure optimal US imaging and minimize surface pressure on the skin, an appropriate amount of gel was applied, and the probe was positioned with minimal pressure to avoid tissue compression while maintaining stable contact for consistent imaging. All US-guided were performed with a Pajunk Sonoplex II 22 G 50 mm needle (Geisingen, Germany). The US machine was placed on one side of the dissection table while the physician stayed on the opposite side, next to the chest wall to be injected. Positions for the operator and the US machine were switched for the contralateral side. Three different color inks were used: black is for the SPIP block with a single injection, green is for the SPIP block with double injections, and blue is for the DPIP block.

#### 2.1.1. Single-Injection Superficial Parasternal Intercostal Plane Block (SPIP)

**Figure 1:**
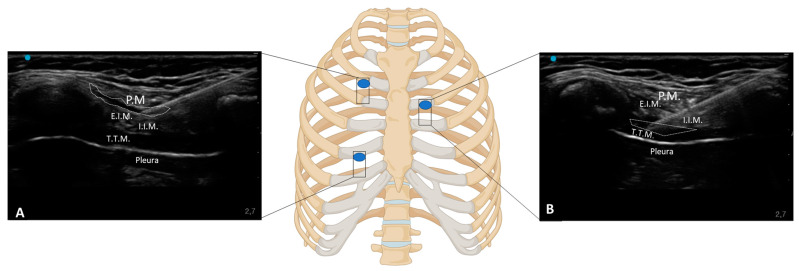
Sono-anatomy of single- (fourth intercostal space) and double-injection (second and fourth intercostal spaces) SPIP and DPIP (third intercostal space) blocks. (**A**) US-guided needle trajectory for the SPIP single-injection and double-injection block techniques, targeting the fascial plane superficial to the external intercostal muscle. (**B**) US-guided needle trajectory for the DPIP block, highlighting the deeper needle placement necessary to reach the fascial plane between the internal intercostal and the transversus thoracis muscles. P.M.: pectoralis major muscle; E.I.M.: external intercostal muscle; I.I.M.: internal intercostal muscle; T.T.M.: transversus thoracis muscle.

The superficial intercostal plane block targets the fascial plane superficial to the external intercostal muscle. Under ultrasound guidance, the transducer was placed longitudinally over the intercostal space at the level between the 4th and 5th ribs, 2 cm laterally from the sternal border. The external intercostal muscle was identified as a hypoechoic layer superficial to the rib. The needle was advanced in-plane from caudal to cranial, targeting the fascial plane superficial to the external intercostal muscle. Ten ml of black ink was injected into this plane, visualized as hypoecheoic spread just above the muscle (Figure 1A).

#### 2.1.2. Double-Injection Superficial Parasternal Intercostal Plane Block (SPIP)

According to Harbell et al. [22], the SPIP block with double injections is similar to the SPIP block and targets the fascial plane between the pectoralis major and the external muscles, 2 cm laterally to the sternum border, at the second and fourth intercostal spaces. The transducer was placed longitudinally along the parasternal line, close to the sternum. The pectoralis major and external muscles were visualized. The needle is inserted in-plane, typically from caudal to cranial and directed towards the fascial plane between these two muscles. The green dye is then injected, creating a hypoechoic spread within these fascial planes. In this setting, the injection of the parasternal plane was performed into two intercostal fascial planes, the second and fifth. Five ml of green ink was injected into each fascial plane (Figure 1A).

#### 2.1.3. Deep Parasternal Intercostal Plane (DPIP) Block

The deep parasternal intercostal plane block involves the deposition of local anesthetic in the fascial plane between the internal intercostal muscle and the transversus thoracis muscle, adjacent to the parasternal region. The US transducer is positioned longitudinally along the parasternal line at the third intercostal level. The internal intercostal muscle and the transversus thoracis muscle are identified. The needle was advanced in-plane, from caudal to cranial, and directed towards the fascial plane between these two muscles. Upon injection, ten ml of blue ink was injected into this plane, visualized as hypoechoic spread just above the muscles (Figure 1B).

### 2.2. Gross Anatomical Dissection

The gross anatomical dissection was conducted 15 min after the end of the three procedures for each side, using the anterior midline incision along the sternum from the manubrium to the xiphoid process. The skin was retracted laterally to fully expose the anterior thoracic wall layer by layer. The muscles (pectoralis major, pectoralis minor, serratus anterior, external intercostal, internal intercostal, innermost intercostal, and transversus thoracic muscles) enveloped by their fasciae were in turn identified and removed by detaching. At this stage, the extent of dye spread was assessed both in craniocaudal and in mediolateral directions. The craniocaudal spreading was described concerning the costal level, and this was explored from the first to the twelfth ribs. At each costal level, the sidelong spread was measured from the edge of the sternum to the point of maximum lateral extension, dividing the chest wall into two zones: medial (between the parasternal line and the midclavicular line) and lateral (between the midclavicular line and the anterior axillary line).

### 2.3. Image Analysis

**Figure 2:**
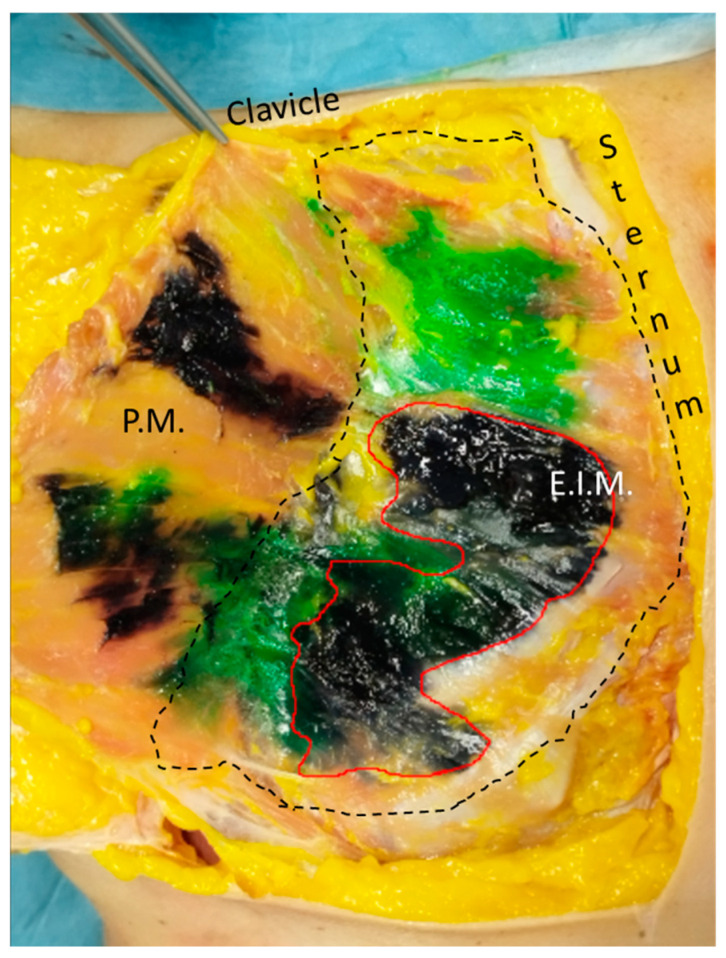
Example of a region of interest at the fascial plane between the pectoralis major and the external intercostal muscles that was delineated around the injection sites, with the extent of ink spread quantitatively assessed in both lateral and vertical dimensions. P.M.: pectoralis major muscle; E.I.M.: external intercostal muscle. Dashed line: fascial plane between the pectoralis major and the external intercostal muscles.

**Figure 3:**
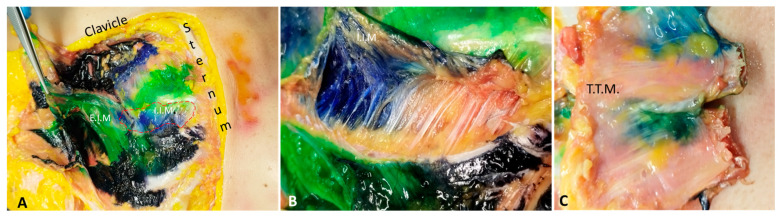
(**A**): Example of a region of interest at the fascial plane between the internal intercostal and the transversus thoracis muscles that was delineated around the injection sites, with the extent of ink spread quantitatively assessed in both lateral and vertical dimensions. Dashed line: fascial plane between the internal intercostal and the transversus thoracis muscles. (**B**): Anterior detailed view of the fascial plane between the internal intercostal and transversus thoracis muscles. (**C**): Posterior detailed view of the fascial plane between the internal intercostal and transversus thoracis muscles, clearly delineating the boundary formed by the transversus thoracis muscle. E.I.M.: external intercostal muscle; I.I.M.: internal intercostal muscle. T.T.M.: transversus thoracis muscle.

Serial photographs were taken using a high-resolution camera to capture the spread of ink in the tissues. A Canon EOS 4000D camera (Tokyo, Japan) with a macro lens was used to capture detailed images of the injection sites and during the dissection layer by layer. Photographs were taken immediately after injections and at 20-min intervals for 3 h. Images were taken from the same angle, perpendicular to the chest wall, to ensure comprehensive coverage of the dye spread area. Standardized settings for aperture, shutter speed, and ISO were maintained to ensure consistency across all images. To morphometrically characterize the pattern of ink spread, the images underwent image processing and analysis using Image J software (version 1.54f, freely available at http://rsb.info.nih.gov/iJ/) [23]. Regions of interest were defined around the injection sites, and the extent of ink spread was measured in both lateral and vertical directions, calculating the areas of spread layer by layer and the distance from the sternum border to the point of the lateral end of the dye spread (Figure 2 and Figure 3).

The evaluated fascial planes were as follows: (1) between pectoralis major and external intercostal muscles; (2) between external and intercostal muscles; and (3) between internal intercostal and transversus thoracis muscles.

### 2.4. Statistical Analysis

Statistical analyses were executed using GraphPad PRISM version 8.4.2 (GraphPad Software Inc., San Diego, CA, USA), adhering to a statistical significance of *p* < 0.05. Normality was assessed via the Kolmogorov–Smirnov test. Descriptive statistics, including measures of central tendency and dispersion (mean ± standard deviation), were computed for each procedure, layer by layer. Comparative analyses between areas of ink spread for each procedure or distances from the sternum were conducted using the Kruskal–Wallis test and Dunn’s multiple comparisons test. The comparison between the right side and the left side was conducted using a paired Student’s *t*-test. Pearson’s correlation test was used to examine the relationship between qualitative and quantitative evaluations.

## 3. Results

### 3.1. Qualitative Assessment

Four sides of two cadavers were injected according to SPIP with a single injection, SPIP with double injections, and DPIP block procedures and subsequently dissected. The cadaveric section did not reveal the significant presence of the three dyes in the subcutaneous tissue, except for minor leakage along the injection pathway. Anteriorly, there was extensive dye spread observed in planes both superficial and deep to the different blocks. The cranio-caudal spread of dye was over many segments and differed across the fascial planes for both sides. Macroscopically, once removed layer by layer, for the fascial planes, as mentioned above, the dye spread ranged and differed among the different blocks.

**Figure 4:**
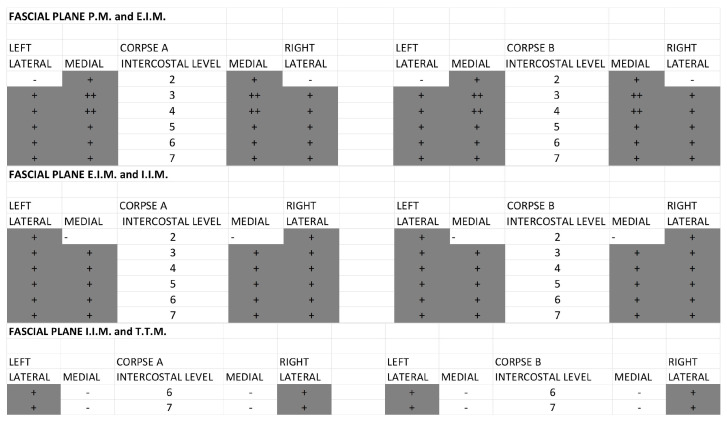
Qualitative assessment: comparing the different fascial planes for the single-injection SPIP block. P.M.: pectoralis major muscle; E.I.M.: external intercostal muscle; I.I.M.: internal intercostal muscle; T.T.M.: transversus thoracis muscle. (+) Positive: presence of black tissue marking dye in the fascial planes. (++) Strongly Positive: strongly presence of black tissue marking dye in the fascial planes. (-) Negative: absence of black tissue marking dye in the fascial planes. For the fascial plane between the external intercostal and internal intercostal muscles, the SPIP had a dye (black) that ranged also from the third to the seventh intercostal space, covering from the medial to the lateral part of the chest wall, while the SPIP block with double injections had a dye (green) spread from the second to the third intercostal space and the fifth intercostal space, medial and one-third of lateral (Figure 5).

For the fascial plane between the pectoralis major and external intercostal muscle, the SPIP with a single injection had a dye (black) that ranged from the third to the seventh intercostal space, covering from the medial to the lateral part, while the SPIP with double injections had a dye (green spread) from the second to the third intercostal space and the fifth intercostal space, medial and one-third of lateral (Figure 4).

**Figure 5:**
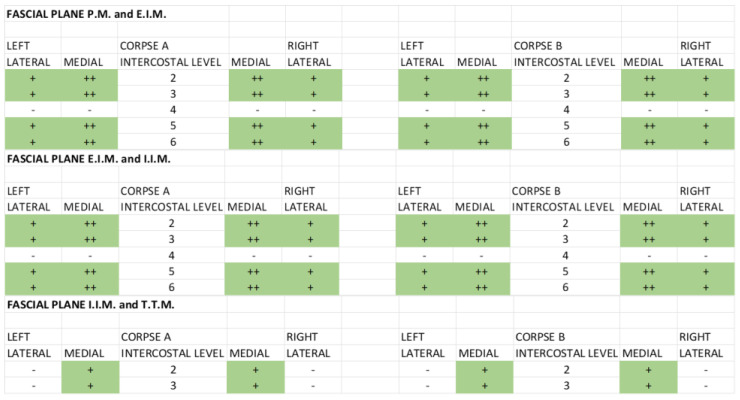
Qualitative assessment: comparing the different fascial planes for the double-injection SPIP block. P.M.: pectoralis major muscle; E.I.M.: external intercostal muscle; I.I.M.: internal intercostal muscle; T.T.M.: transversus thoracis muscle. (+) Positive: presence of green tissue marking dye in the fascial planes. (++) Strongly Positive: strongly presence of green tissue marking dye in the fascial planes. (-) Negative: absence of green tissue marking dye in the fascial planes.

**Figure 6:**
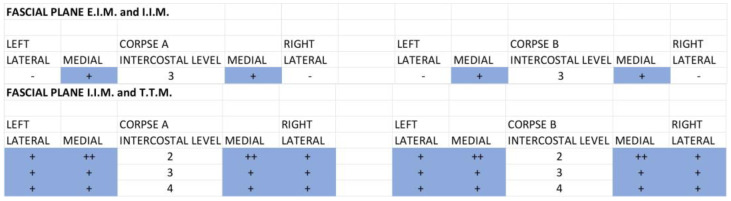
Qualitative assessment: comparing the different fascial planes for the DPIP block. E.I.M.: external intercostal muscle; I.I.M.: internal intercostal muscle; T.T.M.: transversus thoracis muscle. (+) Positive: presence of blue tissue marking dye in the fascial planes. (++) Strongly Positive: strongly presence of blue tissue marking dye in the fascial planes. (-) Negative: absence of blue tissue marking dye in the fascial planes.

For the fascial plane between the internal intercostal and transversus thoracic muscles, the SPIP block with a single injection had no dye spread; the SPIP block with double injections had only the dye spread in the second intercostal space and in the fifth intercostal space, while the DPIP block showed a dye (blue) spread from the fourth to the second intercostal space (Figure 6).

### 3.2. Morphometric Assessment

**Figure 7:**
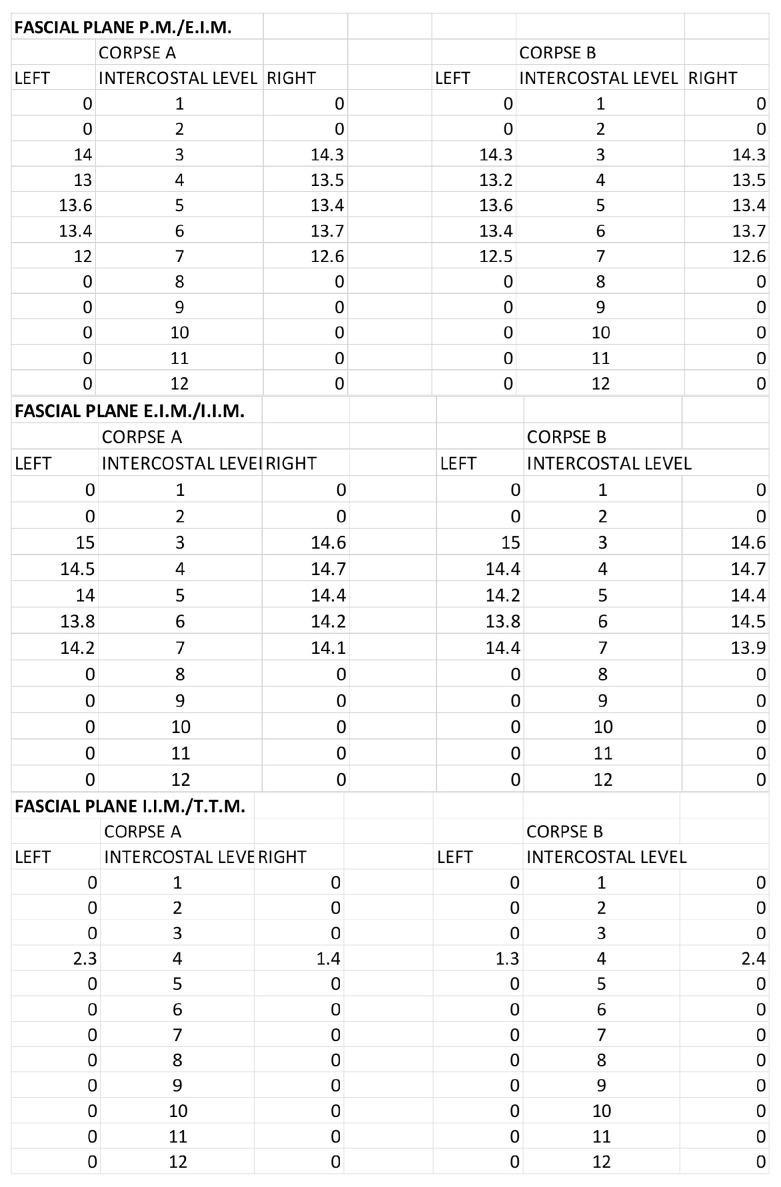
Single-injection SPIP block: Area of ink spread in the different fascial planes. All the measurements are expressed in cm^2^. P.M.: pectoralis major muscle; E.I.M.: external intercostal muscle; I.I.M.: internal intercostal muscle; T.T.M.: transversus thoracis muscle.

**Figure 8:**
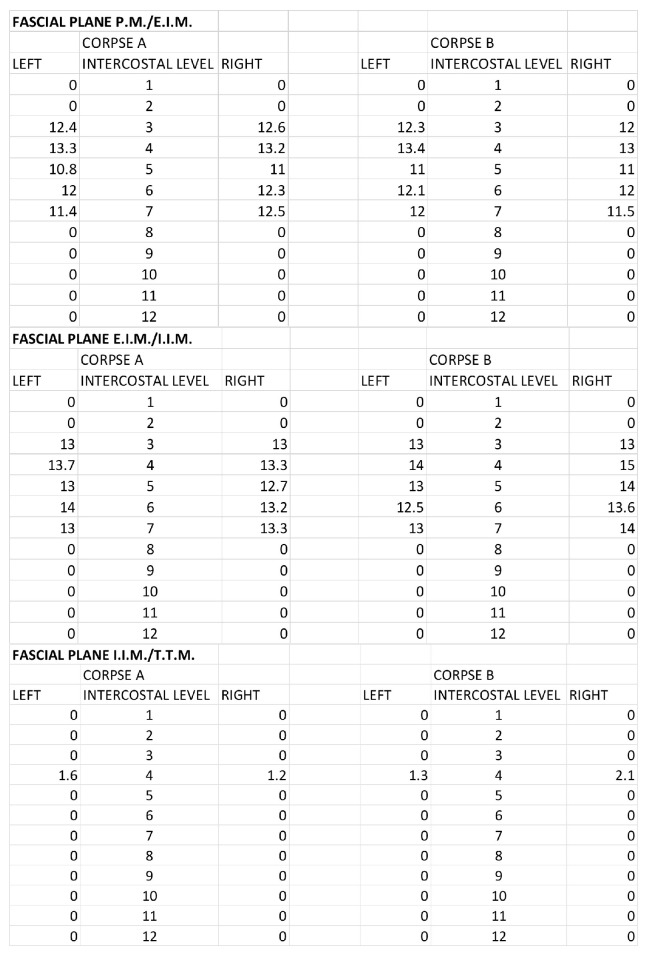
Single-injection SPIP block: distances from the border of the sternum to the point of the lateral end of the color spread. The values are expressed in cm. P.M.: pectoralis major muscle; E.I.M.: external intercostal muscle; I.I.M.: internal intercostal muscle; T.T.M.: transversus thoracis muscle.

The morphometric spread of the dye for both corpses was evaluated by areas of spread layer by layer and the distance from the sternum border to the point of the lateral end of color diffusion. For the SPIP block with a single injection, the area of ink spread and distance from the sternum border are reported in Figure 7 and Figure 8.

**Figure 9:**
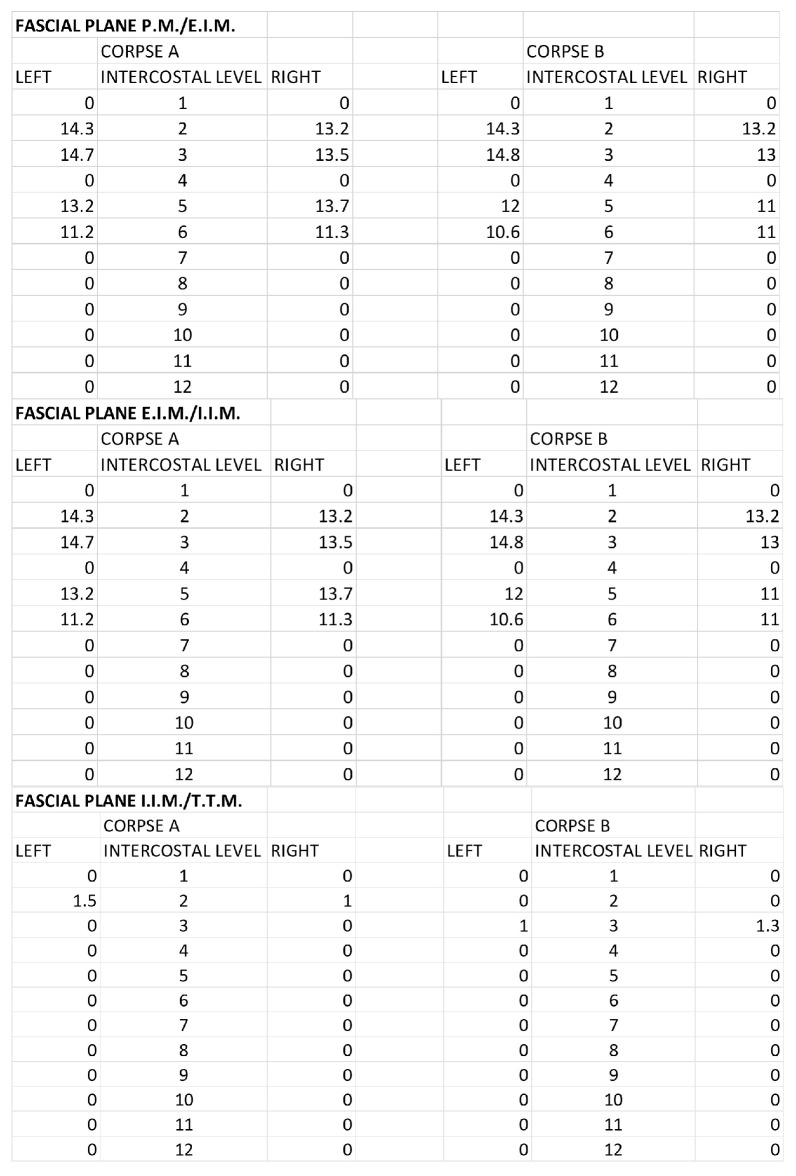
Double-injection SPIP block: Area of ink spread in the different fascial planes. All the measurements are expressed in cm^2^. P.M.: pectoralis major muscle; E.I.M.: external intercostal muscle; I.I.M.: internal intercostal muscle; T.T.M.: transversus thoracis muscle.

**Figure 10:**
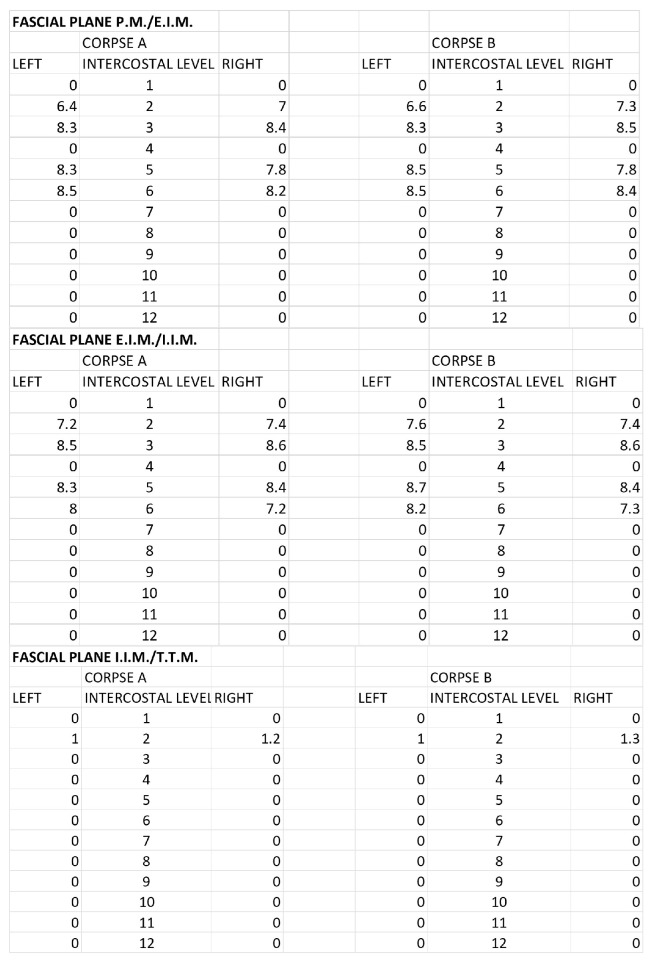
Double-injection SPIP block: distances from the border of the sternum to the point of the lateral end of the color spread. The values are expressed in cm. P.M.: pectoralis major muscle; E.I.M.: external intercostal muscle; I.I.M.: internal intercostal muscle; T.T.M.: transversus thoracis muscle.

For the SPIP block with double injections, the area of ink spread and distance from the sternum border are reported in Figure 9 and Figure 10.

**Figure 11:**
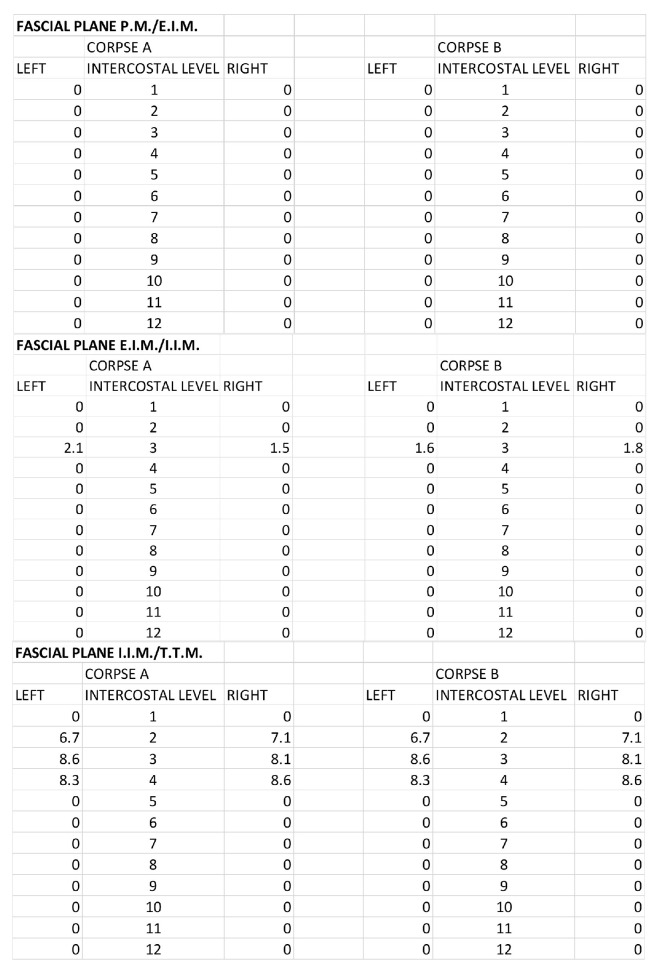
DPIP block: Area of ink spread in the different fascial planes. All the measurements are expressed in cm^2^. P.M.: pectoralis major muscle; E.I.M.: external intercostal muscle; I.I.M.: internal intercostal muscle; T.T.M.: transversus thoracis muscle.

**Figure 12:**
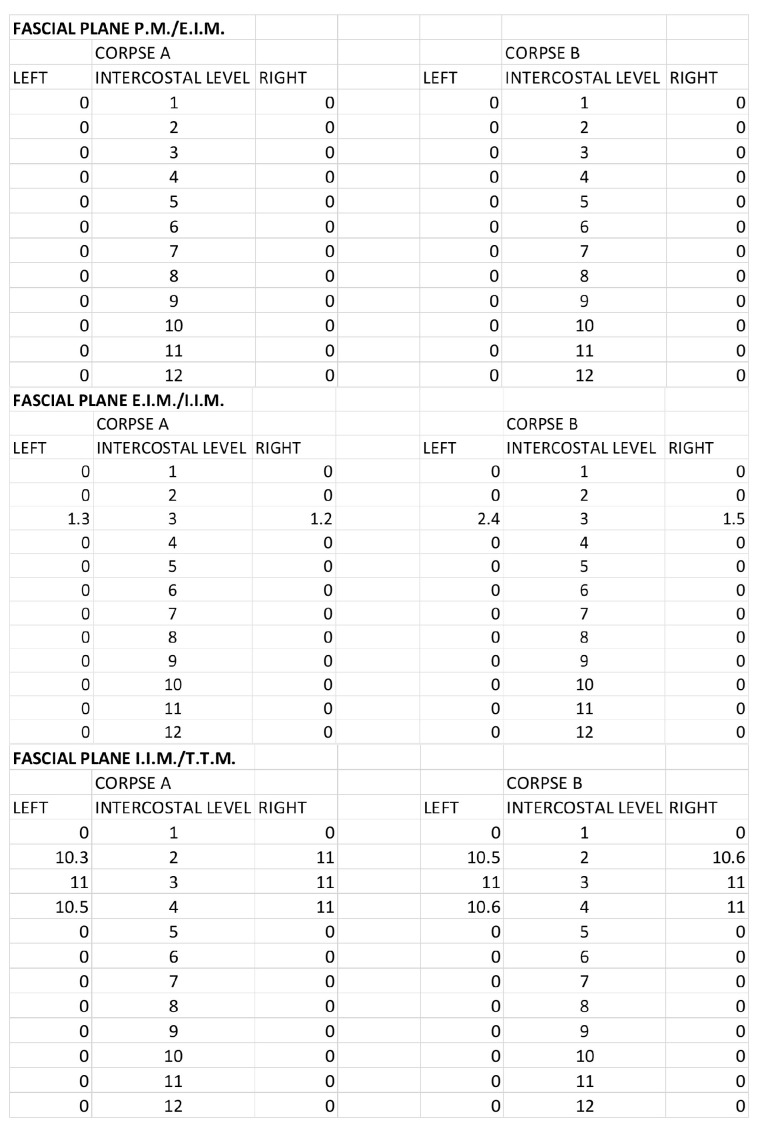
DPIP block: distances from the border of the sternum to the point of the lateral end of the color spread. The values are expressed in cm. P.M.: pectoralis major muscle; E.I.M.: external intercostal muscle; I.I.M.: internal intercostal muscle; T.T.M.: transversus thoracis muscle.

For the DPIP, the area of ink spread and distance from the sternum border are reported in Figure 11 and Figure 12.

#### 3.2.1. Area of Ink Spread: Comparative Analysis Within and Between Fascial Blocks (Single-Injection SPIP Block, Double-Injection SPIP Block, and DPIP Block)

**Figure 13:**
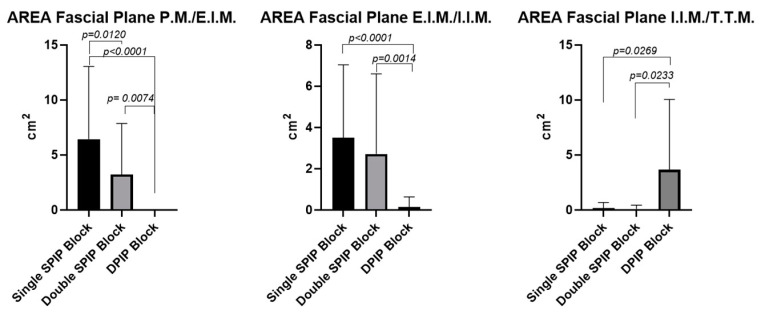
Area of ink diffusion: comparison among the different blocks (double-injection SPIP block, single-injection SPIP block, and DPIP block) within fascial planes. All the measurements are expressed in cm^2^. P.M.: pectoralis major muscle; E.I.M.: external intercostal muscle; I.I.M.: internal intercostal muscle; T.T.M.: transversus thoracis muscle.

According to the paired Student’s *t*-test, no statistically significant differences were observed between the right and the left sides (*p* > 0.05). However, regarding the different fascial blocks, the comparison across fascial planes revealed statistically significant differences in the area of ink diffusion. Similarly, the comparison within the same fascial block also demonstrated significant differences in ink diffusion between the different fascial planes. These differences are reported in Figure 13 and Table 1.

#### 3.2.2. Distances: Comparison Among the Different Planes Within a Single-Injection SPIP Block, Double-Injection SPIP Block, and DPIP Block

**Figure 14:**
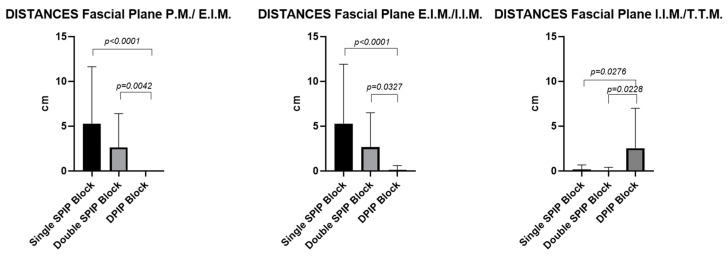
Distances from sternum border: comparison among the different blocks (double-injection SPIP block, single-injection SPIP block, and DPIP block) within fascial planes. All the measurements are expressed in cm. P.M.: pectoralis major muscle; E.I.M.: external intercostal muscle; I.I.M.: internal intercostal muscle; T.T.M.: transversus thoracis muscle.

According to the paired Student’s *t*-test, no statistically significant differences were observed between the right and the left sides (*p* > 0.05). However, regarding the different fascial blocks, the comparison across fascial planes revealed statistically significant differences in the distances. Similarly, the comparison within the same fascial block also demonstrated significant differences in distances from the border of the sternum between the different fascial planes. These differences are reported in Figure 14 and Table 2.

#### 3.2.3. Correlation Area of Ink Spread and Distances from the Border of Sternum

**Figure 15:**
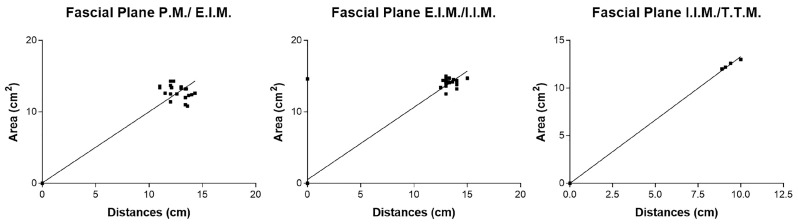
Single-injection SPIP block: correlation area of ink spread and distances from the border of the sternum. P.M.: pectoralis major muscle; E.I.M.: external intercostal muscle; I.I.M.: internal intercostal muscle; T.T.M.: transversus thoracis muscle.

According to the correlation analysis, there was a statistically significant correlation between the area of ink spread and distances from the border of the sternum for all the fascial plane blocks. For the single-injection SPIP block, there were statistically significant correlations in the fascial plane P.M./E.I.M. (r = 0.98; *p* < 0.0001), in the fascial plane E.I.M./I.I.M. (r = 0.86; *p* < 0.0001), and in the fascial plane I.I.M./T.T.M. (r = 1; *p* < 0.0001) (Figure 15).

**Figure 16:**
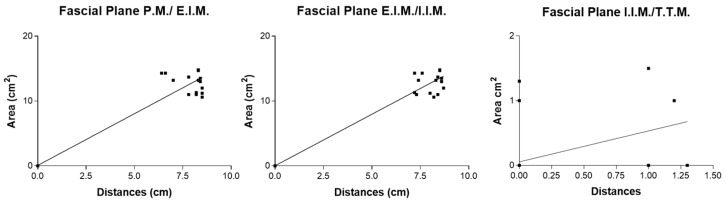
Double-injection SPIP block: correlation area of ink spread and distances from the border of the sternum. P.M.: pectoralis major muscle; E.I.M.: external intercostal muscle; I.I.M.: internal intercostal muscle; T.T.M.: transversus thoracis muscle.

For the double-injection SPIP block, there were statistical correlations in the fascial plane P.M./E.I.M. (r = 0.92; *p* < 0.0001), in the fascial plane E.I.M./I.I.M. (r = 0.98; *p* < 0.0001), and in the fascial plane I.I.M./T.T.M. (r = 0.44; *p =* 0.0016) (Figure 16).

**Figure 17:**
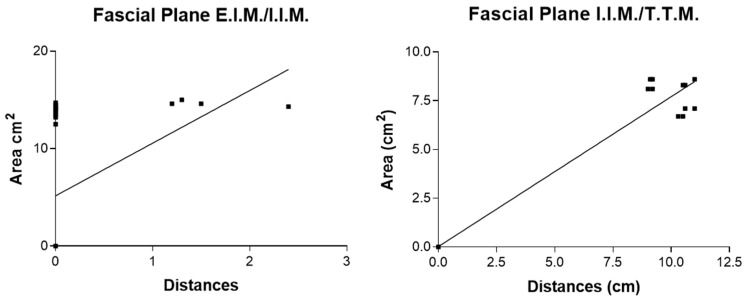
DPIP block: correlation area of ink spread and distances from the border of the sternum. P.M.: pectoralis major muscle; E.I.M.: external intercostal muscle; I.I.M.: internal intercostal muscle; T.T.M.: transversus thoracis muscle.

For the DPIP block, there were statistical correlations in the fascial plane E.I.M./I.I.M. (r = 0.47; *p* = 0.0006) and in the fascial plane I.I.M./T.T.M. (r = 0.98; *p* < 0.0001) (Figure 17).

## 4. Discussion

This is the first cadaveric study to investigate, through gross anatomical dissection, the patterns of local anesthetic spread in single-injection SPIP, double-injection SPIP, and DPIP blocks. By incorporating both qualitative and quantitative assessments, this study offers a comprehensive analysis of ink dispersion dynamics within three distinct fascial planes (P.M./E.I.M., E.I.M./I.I.M., and T.T.M.). The cadaveric dissection revealed minimal presence of dye in the subcutaneous tissue, indicating that the fascial planes effectively contained the injected dye. This suggests that, at least for several hours post-injection, these planes effectively contained the injected dye, maintaining their integrity and preventing significant leakage. This containment likely ensures that the anesthetic remains within the fascial plane, enhancing the efficacy of the block during this time frame. An analysis of our results showed that the number and the location of the intercostal spaces levels revealing macroscopic signs of dye spread were symmetric between both sides for all types of fascial blocks and planes (*p* > 0.05). Throughout the various comparisons, statistically significant differences were shown that were not only numerically compelling (e.g., mean rank differences of 39.67, 28.67, and 20.33 in the inter-fascial block comparisons) but also clinically relevant. These differences revolved around how the injected dye penetrated different fascial layers and how predictably it diffused relative to anatomical landmarks such as the sternal border. A recurring pattern was that the DPIP block showed statistical differences when compared with either the single SPIP block or the double SPIP block. For instance, the largest mean rank differences were observed between the single SPIP block and the DPIP block (39.67; *p* < 0.0001), emphasizing a marked divergence in how the dye spread in these two approaches. This suggested that although a single SPIP block can be precise—when properly administered—it may lack the broad coverage that DPIP appeared to offer in deeper fascial planes. Additionally, double SPIP block vs. DPIP block comparisons yielded significant differences as well, including a mean difference of 20.33 (*p* = 0.0074). These findings suggested that when clinicians select DPIP over SPIP blocks, they are likely to observe a distinct pattern of spread—possibly deeper and different in its distribution characteristics—suggesting that DPIP performs smaller or more controlled ink spread areas. The comparison between single SPIP and double SPIP blocks showed statistical significance only for the P.M./E.I.M. fascial plane, with a mean rank difference of 19.33 (*p* = 0.0120), indicating that the single SPIP block outperformed the double SPIP block in 4.333 (E.I.M./I.I.M.) or 0.250 (I.I.M./T.T.M.) (*p* > 0.05), revealing no meaningful differences in coverage across certain fascial planes. These non-significant results suggested a degree of overlap in the distribution provided by single and double SPIP blocks, indicating that the additional injection does not consistently result in a proportionally greater or more effective spread. Within each fascial block, the data distinguish coverage patterns among the fascial planes: P.M./E.I.M., E.I.M./I.I.M., and I.I.M./T.T.M. In the single SPIP, the fascial plane P.M./E.I.M. vs. fascial plane I.I.M./T.T.M. showed large mean rank differences in injected dye spread (39.00, *p* < 0.0001), underscoring the possibility that the anesthetic spread is more robust in the fascial plane P.M./E.I.M. Moreover, E.I.M./I.I.M. vs. I.I.M./T.T.M. also demonstrates significant comparisons (27.00, *p* = 0.0007), suggesting that incremental anatomical depth might yield highly different diffusion patterns even within the same block. In double SPIP, P.M./E.I.M. vs. I.I.M./T.T.M. did not reach significance (2.667, *p* > 0.05), which implied that a second injection may equalize or redistribute local anesthetic such that differences between these fascial planes were no longer stark. Conversely, E.I.M./I.I.M. vs. I.I.M./T.T.M. showed greater mean rank differences (e.g., 2.667 or 20.41, *p* < 0.01), indicating that the deeper fascial plane still retains unique diffusion features. In DPIP, the comparisons showed modest or non-significant mean rank differences (e.g., −5.500, *p* = 0.7089 for P.M./E.I.M. vs. E.I.M./I.I.M.) but shifted to substantial values when comparing superficial to deeper fascial planes (e.g., −18.50, *p* = 0.0002 for P.M./E.I.M. vs. I.I.M./T.T.M.), such large rank differences reinforce that DPIP’s injected ink can be markedly more (or differently) distributed in deep fascial planes, a point that could be crucial for procedures necessitating coverage in the deeper fascial planes.

The sternum distances comparisons among these fascial blocks (single SPIP, double SPIP, and DPIP blocks) and across distinct fascial planes (P.M./E.I.M., E.I.M./I.I.M., and T.T.M.) showed that single SPIP and double SPIP blocks had no significant differences between P.M./E.I.M. and E.I.M./I.I.M. fascial planes (*p* > 0.05). However, both fascial blocks exhibited, respectively, greater distances in the P.M./E.I.M. (*p* = 0.0009 and *p* = 0.0074, respectively) and E.I.M./I.I.M. (*p* = 0.0004 and *p* = 0.0049, respectively) fascial planes compared to the I.I.M./T.T.M. fascial plane. In contrast, the DPIP block demonstrated no significant difference between P.M./E.I.M. and E.I.M./I.I.M. (*p* = 0.07093) but was significantly greater in the I.I.M./T.T.M. fascial plane compared to the other two (*p* = 0.0002 and *p* = 0.0154). In the I.I.M./T.T.M. fascial plane, cross-sectional analyses revealed that the DPIP block was more adjacent to the sternum than both the single and double SPIP blocks (*p* = 0.0276 and *p* = 0.0228, respectively). In contrast, no significant difference was observed between single and double SPIP blocks (*p* > 0.05).

In qualitative and quantitative assessments, dye spread had a widespread and profound dissemination for a single-injection SPIP block than a DPIP block (*p* < 0.05). The single-injection SPIP block had a huge dye spread in the fascial plane between the pectoralis major and external intercostal muscles, in the fascial plane between the external and internal intercostal muscles, and in the fascial plane between the internal intercostal and transversus thoracis muscles. As has been reported by another recent study [22] examining the dye spread of this block, there was a consistent mediolateral spread from the sternum to the midclavicular line, with several cases showing further lateral extension to the anterior axillary line (Figure 4, Figure 5, Figure 6, Figure 7, Figure 8, Figure 9, Figure 10 and Figure 11). It is noteworthy that while there is communication between the deep fascial planes, this does not extend to the subcutaneous tissue. Furthermore, the inverse is not true; deeper injections do not extend superficially, likely due to differences in compartmental volumes. This suggests that the deep fascia acts as a barrier, preventing upward spread, whereas communication within deep layers allows for a more controlled spread and diffusion of substances like anesthetics within targeted planes.

In the light of these findings, in our fresh frozen cadaveric model, the DPIP block exhibited a more uniform and extensive spread of injected dye along parasternal intercostal spaces compared to SPIP blocks, indicating better coverage of anterior cutaneous nerves when a single injection is administered at the fourth interspace. As the SPIP blocks gain wider acceptance, it becomes increasingly important that clinical research on its efficacy is grounded in methodologies that optimize parasternal spread while minimizing patient risk.

An analysis of study results showed for all the blocks a positive correlation between the distances from the sternum border and the area of dye spread, indicating that as the area of spread increases, so does the distance from the sternum border. For single SPIP, extremely high correlations in the I.I.M./T.T.M. plane suggested an almost perfect linear relationship between injection placement and dye coverage depth. These results implied that in single-injection scenarios, a small adjustment in where the needle is placed along the mediolateral axis could reliably result in a proportional change in spread. For double SPIP, strong correlations existed for P.M./E.I.M. (r = 0.92) and E.I.M./I.I.M. (r = 0.98), both *p* < 0.0001, indicating robust consistency in coverage for superficial and intermediate fascial planes. In contrast, the correlation in the deepest fascial plane (I.I.M./T.T.M.) was lower (r = 0.44, *p* = 0.0016), suggesting that once a second injection site is introduced, the diffusion pattern in the deeper compartment may become less predictable. This variability might be due to the fluid interplay between the two injection points or the more complex fascial architecture encountered at deeper levels. For the DPIP block, they showed a moderate correlation of r = 0.47 (*p* = 0.0006) in the E.I.M./I.I.M. fascial planes., yet a very high correlation of r = 0.98 (*p* < 0.0001) in the I.I.M./T.T.M. fascial plane. This stark contrast suggested that DPIP can be highly effective in targeting deeper structures with consistent coverage. However, at intermediate depths, the relationship between injection distance and dye spread might be subject to additional anatomical or procedural nuances. These results suggest that both the macroscopic and microscopic anatomy of fascial planes play a critical role in either compartmentalizing the anesthetic or facilitating its spread. As has been demonstrated by other studies, there are several factors that can influence the distribution of local anesthetics in fascial plane blocks [24], including the number of injections along the sternum. Clinical studies have varied in their approaches, with some performing injections at each parasternal intercostal space between ribs 2 and 5 [17], while others have used one [25,26] or two injections [27,28,29] per hemithorax.

Additionally, the volume of injected dye and precise orientation of the needle relative to the target fascial planes and nerves are critical in achieving effective spread [24,30]. Moreover, in our results, the single-injection SPIP block in which a 10 mL volume was used resulted in a slightly greater area of dye spread to the double-injection SPIP block and DPIP block. Furthermore, recent studies suggest that there may be microscopic diffusion across fascial planes, potentially reaching additional analgesic targets, further enhancing the block’s effectiveness [24,25,26]. Indeed, when local anesthetics are injected into the fascial plane, they spread through two mechanisms: bulk flow and diffusion [24]. Bulk flow refers to the movement of the injected fluid as a whole through the fascial plane, driven by pressure exerted during injection and related also to the volume. This process, known as hydro-dissection, involves the separation and expansion of the fascial layers. On the other hand, diffusion involves the movement of anesthetic molecules from areas of higher concentration to those of lower concentration within the fascial plane [24]. This process is facilitated by extracellular matrix (ECM), which allows the local anesthetic to spread and reach nociceptors and nerve fibers located within or near the fascial plane. The extent and efficacy of diffusion are influenced by properties of the local anesthetic and the structural characteristics of the fascial plane that regulate fluid dispersion [24]. Indeed, the fascia covering the pectoralis major muscle is a thin layer that attaches medially to the sternum and extends superiorly to integrate with the clavicle’s periosteum. It spans the deltopectoral groove to merge with deltoid fascia superolaterally and continues inferiorly, where it transitions into interconnected superficial fascial layers, among which is the rectus sheath. Between the pectoralis major and latissimus dorsi muscles, this fascia forms the axillary floor, commonly referred to as the axillary fascia [31]. In addition, the intercostal muscles are encased within a specialized fascia that extends over the ribs, forming periosteum. This intercostal fascia creates distinct fascial compartments between the ribs, housing the internal and external intercostal muscles. The intercostal nerves traverse along this fascia, and at the sternum, the intercostal fascia fuses with the periosteum [32]. In light of all this, it is important to note that the spread of injected dye near the parasternal region for DPIP might be restricted by these fascial planes and their attachments, potentially limiting the effectiveness of these blocks. The thick fibrous tissue within the intercostal spaces, along with the fascial attachments, seemingly inhibited the spread of the injected dye. This study therefore indicates that given the distinct anatomical and procedural characteristics of the SPIP (single and double) and DPIP blocks, while DPIP may offer analgesic coverage for the closeness to anterior intercostal nerves, its associated risks may outweigh its benefits in certain clinical scenarios, particularly where the risk of vascular injury is high. On the other hand, SPIP, while safer, may require optimization through multiple injections or combined techniques to achieve the desired analgesic effect in case of trauma, scarring, or fascial densifications that alter the integrity of the compartment. Such conditions could impede the spread of the injected dye, necessitating further technical adjustments to ensure effective outcomes. Moreover, anatomical variations and fusion lines between fascial layers can complicate injections and significantly influence the distribution of local anesthetic [24]. Ensuring adequate volume is crucial, as using a sufficient amount is necessary to promote effective spread through bulk flow [24]. Finally, the positive correlation across all blocks between the distance from the sternal border and the area of dye spread indicates that an increase in spread area corresponds to a greater distance from the sternum. The single-injection SPIP block uses a larger volume injected at a single site, promoting a broader and more continuous spread across multiple fascial planes. This approach is relevant in clinical scenarios where widespread coverage is desired, such as for analgesia following extensive chest wall trauma or surgery. The double-injection SPIP block, on the other hand, divides the total volume between two injection sites, targeting specific fascial planes with greater precision. This technique may be advantageous in scenarios requiring more localized analgesia or in cases where anatomical variations or scarring may impede the spread of injected dye from a single injection.

The SPIP blocks, in particular the single-injection SPIP block, demonstrated the greatest lateral spread, with staining of the medial pectoral nerve occurring in all the specimens and highlighting of an overlap in coverage with the interpectoral (previously PECs I) block. This overlap is anatomically logical, as both blocks are administered within the same interconnected fascial plane. Previous cadaveric studies [33] have shown that when the interpectoral plane block is performed medial to the thoracoacromial artery, it can extend toward the sternum. Additionally, a recent prospective study indicated that patients receiving both interpectoral and pecto-serratus (formerly PECs II) plane blocks for sternotomy analgesia experienced shorter durations of ventilatory support, lower pain scores, and reduced need for rescue analgesia [6,34]. This literature contextualizes the anatomical and procedural findings of this study within the broader framework of fascial plane blocks used for chest wall analgesia. While the SPIP block demonstrated promising lateral spread and nerve staining in our cadaveric model, the integration of complementary blocks like the pecto-serratus may provide additional benefits in certain clinical scenarios, particularly where broader analgesic coverage is required. In summary, while the SPIP block, especially with a single injection, shows potential overlap with interpectoral block coverage, its clinical efficacy and role in multimodal analgesia protocols require further exploration. The anatomical findings presented in this study provide a foundational understanding for future investigations aimed at optimizing the use of SPIP and related fascial plane blocks.

### Limitations of the Study

When employing a cadaveric model to simulate the dissemination of local anesthetic in clinical practice, several significant limitations must be considered [35]. Firstly, postmortem alterations in tissue integrity and permeability may substantially impact dye distribution, potentially leading to a misrepresentation of the anesthetic’s actual spread. Secondly, in living subjects, factors such as intrathoracic pressure fluctuations during respiration, as well as muscle tone and contraction, could result in more extensive dispersion of the anesthetic, which cannot be adequately replicated in a cadaveric model. Thirdly, the positioning of the patient during the procedure plays a crucial role in anesthetic distribution, and this variable may differ between cadaveric and living models. Additionally, the presumption that the observed dye spread can reliably predict the clinical efficacy of the anesthetic remains unproven and necessitates further validation. Furthermore, this pilot study was conducted on only two cadavers, thereby necessitating caution in generalizing the findings due to potential variability of observations. Finally, to account for potential anatomical variability in clinical scenarios, both male and female cadavers were included in the study. While no significant gender-related differences in dye spread patterns were observed, further research with larger sample sizes is warranted to explore this aspect more comprehensively.

## 5. Conclusions

In conclusion, this cadaveric study demonstrated that the single-injection SPIP block resulted in a broader lateral spread of the dye compared to the double-injection SPIP block and DPIP block. These findings emphasize the critical importance of macroscopic and microscopic fascial anatomy, as well as volume optimization, in either compartmentalizing the anesthetic or promoting its distribution, ultimately determining the effectiveness of these blocks.

## Figures and Tables

**Table 1:** Comparison in area of ink spread among the different fascial planes for single-injection SPIP block (S: SPIP), double-injection SPIP block (D. SPIP), and DPIP block. S. = single; D = double. P.M.: pectoralis major muscle; E.I.M.: external intercostal muscle; I.I.M.: internal intercostal muscle; T.T.M.: transversus thoracis muscle. ns: significant; ****: extremely significant; ***: highly significant; *: significant.

Dunn’s Multiple Comparisons Test	Mean Rank Diff.	Significant?	Summary	Adjusted *p* Value
**S. SPIP**				
Fascial Plane P.M./E.I.M. vs. Fascial Plane E.I.M./I.I.M.	12.00	No	ns	0.3032
Fascial Plane P.M./E.I.M. vs. Fascial Plane I.I.M./T.T.M.	39.00	Yes	****	<0.0001
Fascial Plane E.I.M./I.I.M. vs. Fascial Plane I.I.M./T.T.M.	27.00	Yes	***	0.0007
**D. SPIP**				
Fascial Plane P.M./E.I.M. vs. Fascial Plane E.I.M./I.I.M.	5.333	No	ns	>0.9999
Fascial Plane P.M./E.I.M. vs. Fascial Plane I.I.M./T.T.M.	2.667	No	ns	>0.9999
Fascial Plane E.I.M./I.I.M. vs. Fascial Plane I.I.M./T.T.M.	−2.667	No	ns	>0.9999
**DPIP**				
Fascial Plane P.M./E.I.M. vs. Fascial Plane E.I.M./I.I.M.	−5.500	No	ns	0.7089
Fascial Plane P.M./E.I.M. vs. Fascial Plane I.I.M./T.T.M.	−18.50	Yes	***	0.0002
Fascial Plane E.I.M./I.I.M. vs. Fascial Plane I.I.M./T.T.M.	−13.00	Yes	*	0.0154

**Table 2:** Comparison in distances among the different fascial planes for the single-injection SPIP block (S. SPIP), double-injection SPIP block (D. SPIP), and DPIP block. S. = single; D = double. P.M.: pectoralis major muscle; E.I.M.: external intercostal muscle; I.I.M.: internal intercostal muscle; T.T.M.: transversus thoracis muscle. ns: significant; ***: highly signifcant; **: very significant; *: significant.

Dunn’s Multiple Comparisons Test	Mean Rank Diff.	Significant?	Summary	Adjusted *p* Value
**S. SPIP**				
Fascial Plane P.M./E.I.M. vs. Fascial Plane E.I.M./I.I.M.	−1.375	No	ns	>0.9999
Fascial Plane P.M./E.I.M. vs. Fascial Plane I.I.M./T.T.M.	25.00	Yes	***	0.0009
Fascial Plane E.I.M./I.I.M. vs. Fascial Plane I.I.M./T.T.M.	26.38	Yes	***	0.0004
**D. SPIP**				
Fascial Plane P.M./E.I.M. vs. Fascial Plane E.I.M./I.I.M.	−0.8125	No	ns	>0.9999
Fascial Plane P.M./E.I.M. vs. Fascial Plane I.I.M./T.T.M.	19.59	Yes	**	0.0074
Fascial Plane E.I.M./I.I.M. vs. Fascial Plane I.I.M./T.T.M.	20.41	Yes	**	0.0049
**DPIP**				
Fascial Plane P.M./E.I.M. vs. Fascial Plane E.I.M./I.I.M.	−5.500	No	ns	0.7093
Fascial Plane P.M./E.I.M. vs. Fascial Plane I.I.M./T.T.M.	−18.50	Yes	***	0.0002
Fascial Plane E.I.M./I.I.M. vs. Fascial Plane I.I.M./T.T.M.	−13.00	Yes	*	0.0154

## Data Availability

The data presented in this study are available upon request from the corresponding author. The data are not publicly available due to privacy.
